# Nonlinear changes in pupillary attentional orienting responses across the lifespan

**DOI:** 10.1007/s11357-023-00834-1

**Published:** 2023-06-15

**Authors:** Elizabeth Riley, Hamid Turker, Dongliang Wang, Khena M Swallow, Adam K Anderson, Eve De Rosa

**Affiliations:** 1 Department of Psychology, Cornell University, Ithaca, NY USA; 2 Department of Public Health and Preventative Medicine, SUNY Upstate Medical University, Syracuse, NY USA

**Keywords:** Pupillary responses, Aging, Attention, Locus coeruleus, Orienting

## Abstract

The cognitive aging process is not necessarily linear. Central task-evoked pupillary responses, representing a brainstem-pupil relationship, may vary across the lifespan. Thus we examined, in 75 adults ranging in age from 19 to 86, whether task-evoked pupillary responses to an attention task may serve in as an index of cognitive aging. This is because the locus coeruleus (LC), located in the brainstem, is not only among the earliest sites of degeneration in pathological aging, but also supports both attentional and pupillary behaviors. We assessed brief, task-evoked phasic attentional orienting to behaviorally relevant and irrelevant auditory tones, stimuli known specifically to recruit the LC in the brainstem and evoke pupillary responses. Due to potential nonlinear changes across the lifespan, we used a novel data-driven analysis on 6 dynamic pupillary behaviors on 10% of the data to reveal cut off points that best characterized the three age bands: young (19–41 years old), middle aged (42–68 years old), and older adults (69 + years old). Follow-up analyses on independent data, the remaining 90%, revealed age-related changes such as monotonic decreases in tonic pupillary diameter and dynamic range, along with curvilinear phasic pupillary responses to the behaviorally relevant target events, increasing in the middle-aged group and then decreasing in the older group. Additionally, the older group showed decreased differentiation of pupillary responses between target and distractor events. This pattern is consistent with potential compensatory LC activity in midlife that is diminished in old age, resulting in decreased adaptive gain. Beyond regulating responses to light, pupillary dynamics reveal a nonlinear capacity for neurally mediated gain across the lifespan, thus providing evidence in support of the LC adaptive gain hypothesis.

**Supplementary Information:**

The online version contains supplementary material available at 10.1007/s11357-023-00834-1.

## Introduction

Aging is associated with altered regulation of cognitive and bodily function, ranging from memory [51] and attention to autonomic [62] and immune system regulation [12]. The brainstem, which links the brain and the body, contains nuclei critical for regulating activity in both the central and peripheral nervous systems [31]. There is increasing recognition that cognitive aging is related, in part, to degeneration of brainstem nuclei [70], with the locus coeruleus (LC) among the earliest sites of damage on the long road to Alzheimer’s disease (AD) [18, 32, 52, 71, 80]. The LC, which begins to sustain damage even in healthy younger adults [11], has been recognized as a crucial player in the pathogenesis of AD and its structural integrity may act as a possible marker of cognitive reserve, which refers to the cognitive resilience in the presence of AD pathology [14]. It sends noradrenergic projections to the central and peripheral nervous systems and plays a major modulatory role in regulating attention and physiological arousal [8, 58, 70]. While pathological changes to the LC are thought to accelerate cognitive decline due to direct loss of noradrenergic LC neurons [32], it is unclear how to characterize changes in the healthy aging brain. Here we examine peripheral pupillary dynamics related to central attentional orienting as a putative biomarker of LC function [25, 28] and its susceptibility to neurotypical aging across the lifespan.

The pupillary window into LC function is the result of the LC’s regulation of the autonomic nervous system, and specifically its control of pupillary dynamics. The pupil not only reflexively responds to changes in external luminance, but also is regulated by neurocognitive activity [22]. Therefore, cognitively driven or task-related pupillary responses are a visible marker of engagement of central resources of putative LC origin. While sympathetic stress, which supports a fight or flight response, results in robust pupil dilation, mundane conditions can also influence these pupillary dynamics, potentially reflecting waxing and waning demands on the reticular activating system that supports ongoing adjustments in alerting and orienting [24, 27, 75]. This brainstem-pupil relationship affords an indirect observation of cognitive processing [9, 13, 20, 74]. Previous work has confirmed that cognitive task-related LC activity is strongly correlated with pupil diameter [16, 20, 39, 54].

Aging has an evident effect on peripheral pupillary responses to light: reduced overall pupil size, diminished darkness reflex, and prolonged light reflex recovery time are consistent with a peripheral sympathetic deficit, and overall dampening of pupil dilation [10] is consistent with age-related peripheral miosis [83]. There is evidence that central task-evoked pupillary responses (TEPR), as indexed by dynamic changes in pupil diameter, may also change throughout the lifespan. Although there is not yet a systematic characterization of the relationship, such studies have provided evidence that TEPRs are inconsistently related to age [17, 28, 36, 55, 76].

Discrepancies across studies could be the result of a few issues that complicate the measurement of TEPRs in older adults. First, the magnitude and direction of TEPRs are very likely dependent on the specific cognitive demand. Some studies have used working memory tasks resulting in dynamic cognitive load, associated with varying task difficulty [20, 25, 40]. Others have used vigilance/alerting tasks [54, 74] that require a more constant cognitive load. However, even within alerting tasks, there is evidence that the strength of the alerting stimulus itself can result in differential responses between age groups [28]. As a task increases in cognitive complexity, it necessarily draws upon brain networks well beyond the brainstem and LC. To better characterize potential age-related changes in brainstem contributions to pupillary dynamics, it may be best to focus on the basic orienting function of the LC [59], which limits cognitive demands. Such data may also illustrate the importance of involuntary pupillary responses in characterizing the time course of aging beyond traditional volitional behavioral measures of accuracy and response time.

Another critical aspect to characterizing age-related contributions of the brainstem to pupillary dynamics is appropriately normalizing for age-related differences in peripheral pupillary dynamics [10]. While the issue of normalization is relevant to younger adults, it is most critical when looking for age differences. Because of age-related miosis [37, 45, 83], older adults do not dilate the pupil as much as younger adults in response to light or cognitive stimuli [10, 17, 81]. While the origin of this pupillary dampening is uncertain, studies suggest that it may be either a consequence of altered central neuromodulatory influences or a weakness in the dilator pupillae muscle in the periphery (see [37, 83]). This narrowing of pupil size may itself be compensatory, trading off dynamic range for acuity and correcting for optical aberrations. For these reasons, simple subtractive normalization, i.e., subtracting a pretrial baseline, may be inadequate for older adults [56]. Accordingly, a more appropriate approach may be to assess, in each participant, not only the available dynamic range of the pupillary response but also what proportion of the available dynamic range is used to support normal orienting [72]. Decreasing pupillary dynamic range across the lifespan functionally limits the ability to discriminate signal from noise. How much of one’s available range is utilized in orienting to stimulus events may be a critical marker of compensatory changes related to reduced signal-to-noise ratio in perceptual processing during cognitive aging [6, 60, 63].

On top of the need for appropriate normalization and scaling, when looking at pupillary responses across the lifespan, it is also important to consider that changes in pupillary behavior may not be limited to “old age” as traditionally defined. A limiting factor in comparing across studies is the use of traditional age groupings. Rather than a priori but relatively arbitrary age bracket boundaries, a data-driven approach to group definition may better characterize a biomarker of changing brainstem contributions across the lifespan. Collapsing across heterogeneous age groupings can mask important differences and potential nonlinearities in function across the lifespan [26]. There is a need for a data driven definition of brainstem aging; pupillary orienting and a characterization of its associated spatiotemporal features may serve as a new approach to understanding how aging progresses nonlinearly across the lifespan and discontinuities that may define age “bands” [26].

Emerging evidence suggests that pupillary responses in midlife are predictive of cognitive outcomes [40]. This is consistent with the fact that pathology in the LC begins decades before symptoms of neurodegenerative disease occur [5], and also with the large body of evidence showing that health status and behaviors in midlife are correlated with cognitive aging outcomes [42]. Indeed, midlife may be the most critical time during which future pathology can be prevented. There is evidence in many fields of cognition that young-old and old-old adults differ in aspects of cognition [23]. On top of this, there is already some evidence that LC composition [49, 61] and connectivity [33] follow nonlinear trajectories across the lifespan with significant differences between younger, middle aged, and older adults. For this reason, it is crucial not only to evaluate pupillary responses in mid-life but also to carefully consider how age group comparisons are made, because there is strong reason to believe that the LC functions differently across the lifespan.

Finally, to address the LC and its role in modulating pupil diameter, we must consider its most basic function—orienting. Pupillary dynamics and eye aperture regulate visual sensitivity, i.e., gain [46], in response to behaviorally relevant stimuli, where increased dilation permits increased light to enter. Consistent with the adaptive gain theory of LC function [3], orienting tasks, in particular, have been shown to reliably engage the LC to elicit phasic pupillary responses as part of the orienting response [43, 79]. Our putative LC-mediated phasic orienting task [59, 64, 65, 68] was a simple auditory discrimination task that produces pupillary responses related to discriminating behaviorally relevant tones from equiprobable behaviorally irrelevant distractor tones, as well as baseline trials [67]. This orienting task is advantageous because it is (1) auditory rather than visual, (2) does not require any shifts in eye gaze or spatial attention and thus is more likely to be associated with the LC, as opposed to other brainstem regions [79], and (3) has also been shown to be independent of manual motor response [66, 67].

In summary, we measured brief, task-evoked phasic pupillary orienting to behaviorally relevant targets and irrelevant distractors. Existing theories of LC functioning can help to frame potential outcomes. Consistent with the adaptive gain theory [1], pupillary orienting to information may follow a monotonic aging trajectory, reflecting increasing cognitive demands of progressive cognitive aging, and thus increasing need for LC gain in order to maintain performance. According to the cognitive reserve theory of LC function and cognitive aging [14, 57], simple pupil orienting responses may index otherwise behaviorally silent changes in brain function, but may be rendered at some point unsustainable. As overall gain in the LC is increased in order to compensate, as required, adaptive gain may be reduced. Eventually, even overall increased gain may not be sustainable, resulting in both decreased gain and adaptive gain in old age.

## Methods

### Participants

We examined 87 participants between the ages of 19 and 86 who completed this task as a part of a larger study involving structural and functional magnetic resonance imaging (MRI) scans, neuropsychological assessment, and other measurements. Pupillometry was not available from 9 participants due to equipment malfunction or inadequate data quality, and in certain older adults, inability to track the pupil due to severe ptosis or the presence of artificial lens implants.

### Participant characteristics

Our final data set included 75 adults, mean age = 48.9 years, SD = 21.3 years, range = 19–86. The definition of age categories is described in the “Defining age groups” section. Participants were screened for diagnosed cognitive impairment, neurological disease, head injury, ocular disease, and had vision and hearing that were normal or correctible to normal. All were fluent speakers of English. Younger, middle aged, and older adults had an average of 17.7 years (SD = 3.5), 16.4 (SD = 3.3), and 17.5 years (SD = 2.8) of education respectively; this did not differ between groups, *F*(2,72) = 1.27, *p* = 0.28.

### Task overview

Participants were asked to remember a series of pictures while performing a go-no go auditory discrimination task. Participants listened for two types of tones (low and high) and responded by pressing a button for the target tone, but not the distractor tone. Participants completed 4 blocks of the task with the identity of the target switching each time.

### Task stimuli

Tone stimuli were either high (1200 Hz) or low (400 Hz) and were 60 ms duration. Background visual stimuli were presented to maintain a consistent level of luminance and cognitive engagement across the testing session. They consisted of 144 color pictures and were evenly divided among pictures of faces, objects, and scenes. We generated an additional 144 scrambled image masks derived from the source images. The images were acquired from online resources [29, 30,84http://vision.stanford.edu/projects/sceneclassification/resources.html] and personal collections. Between trials, the scrambled masks were presented to maintain light stimulation and were created by dividing an image into 256 squares and randomly shuffling them. Pixel intensities, both mean and variance, were matched across images using the SHINE toolbox [82]. For examples of task stimuli, see Supplementary Fig. 1.

### Task procedure

All participants performed the task as part of a longer MRI protocol. Each participant completed 4 blocks of 6 min 47 s each, for a total duration of less than 30 min, with brief breaks. On each 1.25 s long trial, one image (7 × 7 visual degrees; 256 × 256 pixels) was presented for 625 ms and immediately followed by a scrambled version of that same image for another 625 ms. This timing, with no blank screen between trials (0 ms), encouraged vigilance and rapid response times to help equate performance in younger and older participants.

On task trials (144 per block), participants first saw a picture and then a scrambled version of the same picture. Task trials were designated as target, distractor, or no tone trials in equal numbers. Participants were instructed that memory for the pictures would be tested later to ensure they attended to all aspects of the task. Participants were asked to maintain fixation on a dot (0.25 visual degree diameter, red) at the center of the picture throughout the testing session. All 144 images were presented one time per block for a total of 4 repetitions across blocks and 576 total task trials. On task trials, either a high- or low-pitch or no tone played. Participants were told which was the target tone pitch, and this alternated across blocks, with starting target tone counterbalanced across participants. When participants heard the specified target pitch for that run, participants pressed a button with their dominant hand pointer finger. Participants were instructed to make no motor response on trials with a distractor tone or no tone. Before the experiment, participants practiced the task. Tone volume was adjusted during a mock scan to ensure that participants were able to hear the tone over scanner noise. Tone sound level was always set to a standard to begin with and was raised only if participants were not able to discern the two different tones, with sound level ranging between 89 and 92% of maximum across participants.

From the perspective of the participant, there was a constant stream of scrambled images interspersed with intact pictures. Isoluminant changing and distinct background scrambled images, 164 per block without sound, were the majority of events to promote relatively constant low-level visual stimulation for pupil response measurement. These 164 scrambled images were in addition to the 144 pictures associated with trials and the 144 scrambled masks of each that followed it. The additional scrambled images also served to increase the unpredictability of the task trials and enhance the separation of the temporal profile of pupillary responses. The median interval between true non-scrambled task trials was 2.5 s.

The orienting task had a 3 × 6 design, with within-subject factors of tone type (no tone, distractor tone, target tone) and image type (female face, male face, beach, forest, car, chair); the latter included to examine potential image category effects. The trial sequence, specifically, the order and timing of each of the 18 trial types, was optimized using the AFNI function *make_random_timing* to produce sequences that maximized orthogonality of overlapping pupillary responses across trials and minimized the amount of unexplained variance in a simulated task. Inter-trial intervals were filled with scrambled images, as described above.

### Pupillometry

Eye movements, blinks, and pupil size were recorded with an Eyelink 1000 Plus MRI Compatible eye tracker (SR-Research, Ottawa, Ontario, Canada) focused on the right eye using mirrors, at 1000 Hz sampling rate. The eye tracker was calibrated just prior to task onset, using a 9-point calibration routine and manual adjustment of contrast. Calibration was re-checked before each block. During all runs, participants were reminded to blink only when necessary for comfort and center their gaze on a fixation dot.

### Pupil data processing

Raw pupil data for each participant and block were formatted using the EyeLink DataViewer application (SR-Research, Canada) and then cleaned using custom routines. Linear interpolation was used to estimate pupil size during any blinks flagged by EyeLink software, plus inside a 50 ms margin before and after the blink. Since blink artifacts were still present after filtering those automatically flagged, remaining blink artifacts were then removed, with margin (and subsequently interpolated), by thresholding the data using a formula recommended by Kret and Sjak-Shie [41]: (median normalized dilation speed) + 8 * (median absolute deviation). The factor of 8 was chosen empirically to fit our needs as recommended by Kret and Sjak-Shie. Following removal of blink artifacts, data was smoothed with the "rloess” method. Corrections for eye movement were not made because participants fixated on a central point during the entire task and eye movement was confirmed to be minimal. Visual inspection confirmed that artifacts had been appropriately handled by the algorithm.

After cleaning, participants’ dynamic range was then estimated by calculating the difference between the 1st and 99th percentile clean pupil diameter values for that individual across the entire experimental time series. Pupil size histograms were examined for each participant to ensure that each had an approximately normal distribution, and that the 1st and 99th percentile values were not artifactual. Then, tonic pupil size (pretrial) and phasic (trial-evoked) pupil response metrics were calculated for each individual trial. Pretrial baseline pupil size was defined as the mean pupil size in the 500 ms window preceding the trial (derived as the average across pretrial 500 samples). All values were normalized by subtracting that pretrial baseline size. For dynamic range normalization, pupil size values were scaled by dividing by each individual’s dynamic range. Dynamic range normalized results are presented in the main text; see Supplemental results normalized by pretrial baseline only. Phasic pupil response parameters were calculated for the first 2 s following trial onset (derived from 2000 trial samples), where the expected peak of the response was approximately 1 s [67]. Based on published recommendations, parameters considered included area under the curve (AUC, trapezoidal method), maximum, latency to maximum (time in ms past trial onset at which maximum pupil diameter occurred), and maximum positive and negative rates of change [41]. For visualization purposes, average pupillary response curves were also calculated for each group expressed in proportion of dynamic range.

### Statistical analysis

#### Dummy coding

In analyses in which tone type (no tone, distractor, or target) was a predictor of outcomes, no-tone trials were coded as 0, distractor as 1, and target as 2. For age groups, younger adults were coded as 0, middle-aged adults as 1, and older adults as 2.

#### Models

Linear mixed effects models were run in R using the lme4 package [4]. All trials for all individuals were included in the models. Summary statistics, including *p* values and *F* statistics, were calculated using likelihood ratio tests with the *anova* function (or the *joint_tests* function in the case of generalized linear mixed effects models) and are reported in the text. Complete result tables including *t* statistics and confidence intervals were produced using the Wald method with the sjPlot package function *tab_model* [50]. *P*-values were adjusted using the Holm method. Post-hoc interaction and estimate plots were made using *plot_model* command from the sjPlot package.

## Results

### Defining age groups

Our first question was how to group our participants such that variations in the lifespan trajectory of pupillary dynamics were best represented. To capture this variation, we used a grid search to define 3 age groups based on phasic pupillary responses. In a grid search, a parameter space is exhaustively searched to determine optimum values. First we selected a random 10% of target trials (19 trials from each participant). These trials were used for group definition only and not analyzed later. Submitting only target trials further afforded independence from specific hypothesis tests related to other trial types. For each trial, 6 pupil response parameters (AUC, maximum, minimum, latency to maximum, maximum positive rate of change, and maximum negative rate of change) were entered into a principal component analysis (MATLAB function *pca*). The principal component analysis revealed that 99.99% of variance was captured by the first two principal components, which depended almost exclusively on the metrics of latency to maximum and AUC (Table 1). The principal component scores (expression of the original inputs in principal component space) for these first two principal components were then summed for each individual trial, resulting in a single number per trial. These scores were then entered into a grid search to identify the boundaries of three age groups such that linear fits to each of the three groups resulted in the maximum *R*-squared overall. Group definitions were limited to a first break anywhere between ages 18 and 50 and a second break anywhere between ages 55 and 75. The number of groups was set at 3 to ensure an adequate number of individuals in each group. Ultimately, the age groupings that resulted in the best fit were 18–41, 42–68, and 69+ . After defining our three age groups as younger (19–41 years of age, *n* = 31), middle aged (42–68 years of age, *n* = 29), and older (69–82 years of age, *n* = 15), these groupings were used for all analyses and were applied to independent sources of data (remaining 90% of trials). This allowed a closer characterization of orienting pupillary dynamics that may assist in revealing age-related boundaries in brainstem function.

**Table 1:** Result of principal component analysis on six pupil characteristics (AUC, maximum, minimum, latency to maximum, maximum positive derivative, maximum negative derivative). Left table shows variable loadings on each of the resulting principal components. Right table shows the percent of variance explained by each principal component. This analysis used dynamic range normalized data

	Principal component loadings						
Variables	1	2	3	4	5	Component	% explained
AUC	0.16	0.99	< 0.001	< 0.001	< 0.001	**1st**	90.82
Maximum	< 0.001	< 0.001	− 0.49	0.87	− 0.045	**2nd**	9.81
Minimum	< 0.001	< 0.001	0.87	0.49	0.027	**3rd**	< 0.001
Latency to max	0.99	− 0.15	< 0.001	< 0.001	< 0.001	**4th**	< 0.001
Max. pos. Deriv	< 0.001	< 0.001	− 0.035	0.018	0.84	**5th**	< 0.001
Max. neg. Deriv	< 0.001	< 0.001	0.024	− 0.024	− 0.54	**6th**	< 0.001

### Neurocognitive assessment

All participants were screened for cognitive impairment with the Montreal Cognitive Assessment. Younger adults had an average score of 28 (range 25–30, 1 below cutoff), middle-aged adults had an average score of 26.8 (range 19–30, 7 below cutoff), and older adults had an average score of 26.9 (range 20–30, 2 below the cutoff). None had a diagnosis of cognitive impairment of any kind. Participants were also given the Trail Making Test Part B, with an average time of 68.9 s (SD = 23.6, range 40–119) for younger adults, 80.7 s (SD = 58.7, range 39–275) for middle-aged adults, and 84.9 s (SD = 43.6, range 50–237) for older adults. Due to large variability within each group, these scores did not differ significantly, *F*(2,72) = 0.86, *p* = 0.42, see Supplementary Fig. 2.

### Performance on orienting task

#### Accuracy

**Fig. 1:**
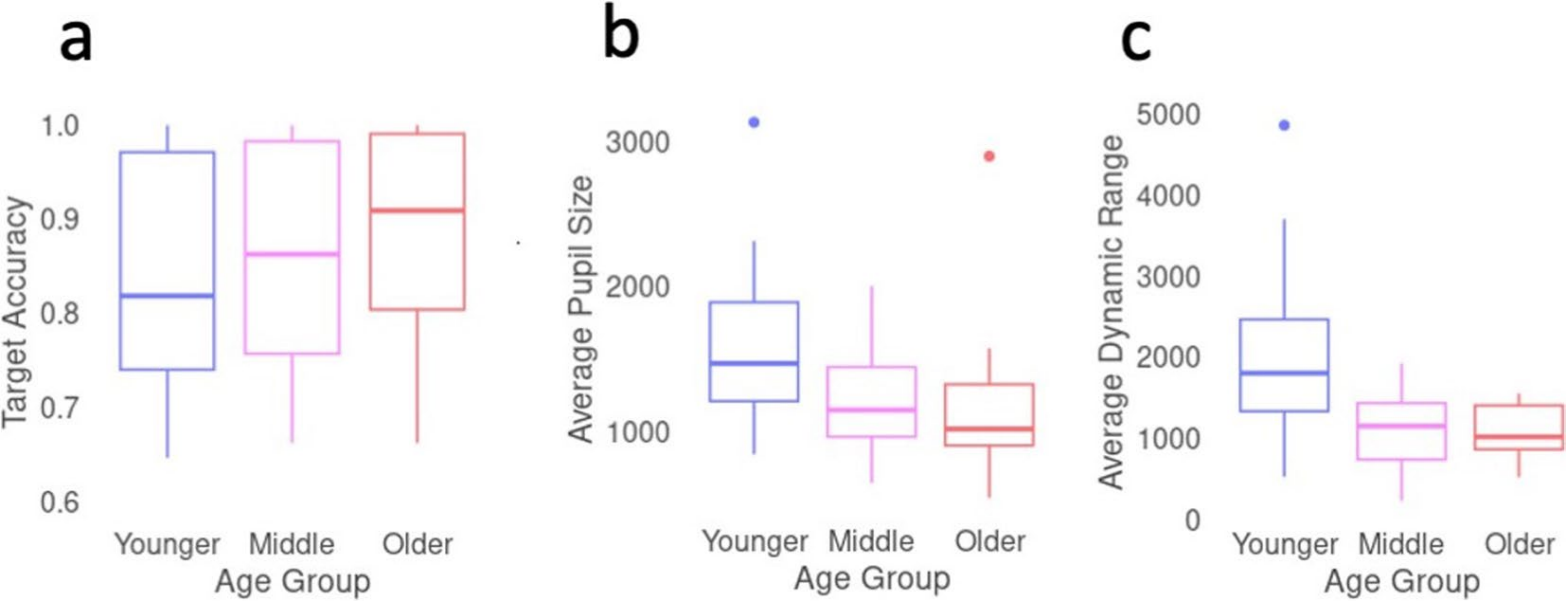
**a** Task accuracy (proportion of targets receiving a button press) by age. **b** Average pupil size throughout the entire experiment by age. **c** Dynamic range (difference between 99th percentile and 1st percentile pupil diameter values after data cleaning) by age. The top and bottom of the box show 25th and 75th percentiles with the median indicated by the middle line. Whiskers show smallest and largest non-outlier values, individual points indicate outliers

Overall, there were no significant age differences in performance on the tone discrimination task. We ran a mixed effects model [4] to model the effects of age group and tone type on task accuracy, with a fixed effect of age group (younger, middle aged, older), and random effects of image and participant. There was no significant effect of age group on response accuracy, *F*(2,inf) = 0.07, *p* = 0.93, although there was a trend toward better accuracy in the older adults (Fig. 1A).

#### Response time

Only response times faster than 1250 ms were considered valid because later responses might have been false alarms in response to a subsequent trial. Response times were calculated on accurate trials only. Younger participants responded to target tones in an average of 402 ms (SD = 132), middle aged in 402 ms (SD = 121), and older adults in 433 ms (SD = 143). This ~ 30 ms age difference in response time in the older group was significantly different from middle age, *t*(3131.2) =  − 6.56, *p* < 0.0001.

### Pupillary dynamics

#### Baseline pupil size

We first evaluated whether the three age groups had similar quality data. All groups required similar amounts of data interpolation during the cleaning process, 24.8%, 13.1%, and 21.1%, respectively, in younger, middle-aged, and older adults. There was no significant difference in proportion of data interpolated, *F*(2,71) = 2.69, *p* = 0.07. Most interpolation was due to blinks while in the MRI scanner, while some could be attributed to the difficulty of obtaining steady pupil measurements. This was a modest increase above the expected 10–15% loss due to blinking alone [7] and is attributable to the fact that it is necessary to trim the margins of blinks to ensure high-quality data [41].

We next checked for age group differences in average pupil size and dynamic range. Consistent with age-related miosis, average pupil size decreased with age, with middle-aged and older adults having an average pupil size 80% and 73% as large as younger adults, respectively. Using a one-way ANOVA to compare individual average pupil sizes between age groups, the difference was significant, *F*(2,71) = 4.88,* p* = 0.01, with both middle-aged and older adults having smaller baseline pupil size than younger adults (Fig. 1B). More pronounced than average pupil size, pupillary dynamic range also decreased with age, with middle-aged and older adults having a dynamic range 59% and 53% as large as younger adults, respectively (Fig. 1C), *F*(2,71) = 13.6,* p* < 0.001, with middle-aged and older adults having compressed dynamic range compared to younger adults.

To take this differential dynamic range into consideration, we conducted our pupil analyses according to two methods: first using the pretrial baseline normalized data and second after also using dynamic range normalization in addition to pretrial baseline normalization. Normalizing data using the pretrial baseline does not take into account any putative peripheral causes of age-related miosis such as weakness in the dilator pupillae muscle [37]. By contrast, dynamic range normalization presents a view of pupillary dynamics concerned with how much internal gain is applied to an age-dampened pupillary response, which more likely reflects central contributions. For this reason, our primary focus was on dynamic range normalized data.

#### Phasic pupillary responses

**Fig. 2:**
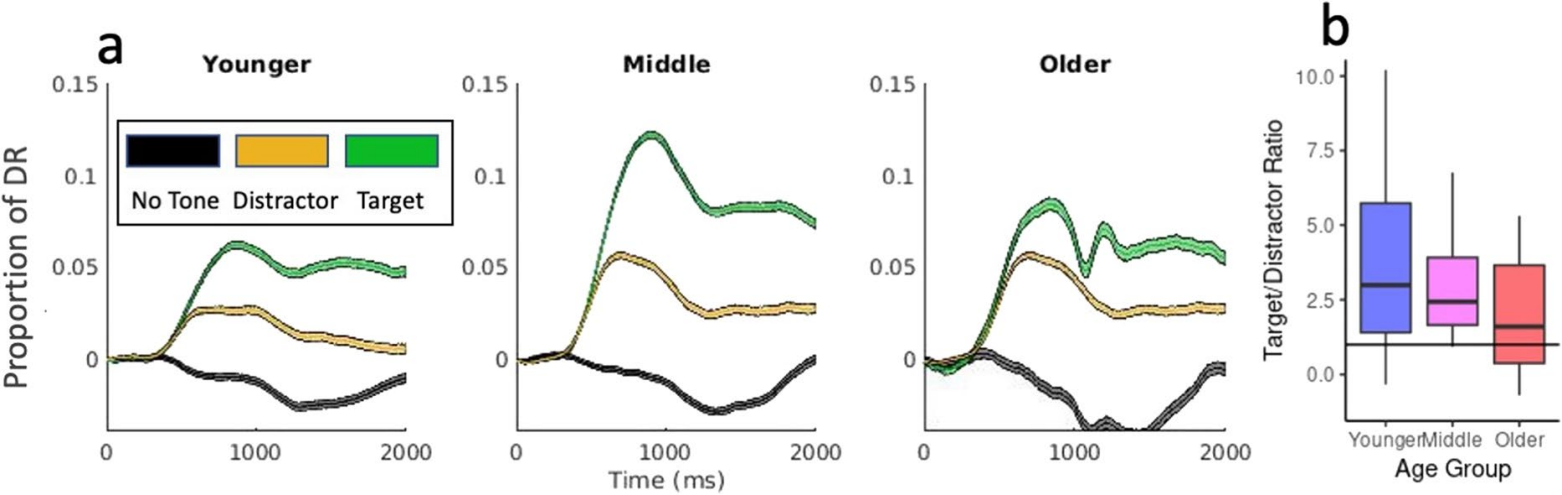
**a** Pupillary response curves in response to no-tone (N, black), distractor (D, orange), and target (T, green) trials in younger, middle aged, and older adults after dynamic range normalization. The colored ribbon shows standard error of the mean. **b** Scores showing the average ratio between target AUC and distractor AUC, by individual, for each age group. The top and bottom of the box show 25th and 75th percentiles with the median indicated by the middle line. Whiskers show smallest and largest non-outlier values. DR is dynamic range

To examine the effects of age and trial type on pupillary responses, we plotted average pupillary response curves for no-tone, distractor, and target trials in all three age groups (Fig. 2A and Supplementary Fig. 3). Overall, all age groups show pronounced differentiation in pupillary responses based on trial type, with largest and longest responses to target trials, smaller responses to distractor trials, and slight pupillary constriction (below pretrial baseline) in response to no-tone trials. There was also evidence of a biphasic response profile, with an initial phasic orienting to the tone trials that rapidly subsided to a more sustained response. The orienting response to both target and distractor tones had a similar onset, with their differentiation beginning after approximately 150 ms. This is consistent with an initial orienting to both tones, but with amplitude and latency to peak amplitude discriminating the unique behavioral relevance of targets. The response curves have a similar shape across age groups, but differ in amplitude, with middle-aged adults using approximately twice as much of their dynamic range as younger adults for an average response.

#### Pupillary response characteristics

We next characterized trial-specific pupillary responses in a 2 s window following event onset by calculating the area under the curve (AUC), maximum pupil diameter, and latency to the pupil maximum. Two final measures, maximum positive and negative derivatives of pupil size, were calculated to capture the rate of change in pupil size. Analysis of all of these characteristics, with both types of normalization, is presented in Supplementary Tables 1 and 2. However, our principal component analysis demonstrated that variance in phasic pupillary responses is captured by the two variables AUC and latency to maximum. Therefore, we present inferential analysis focusing on these two variables.

**Fig. 3:**
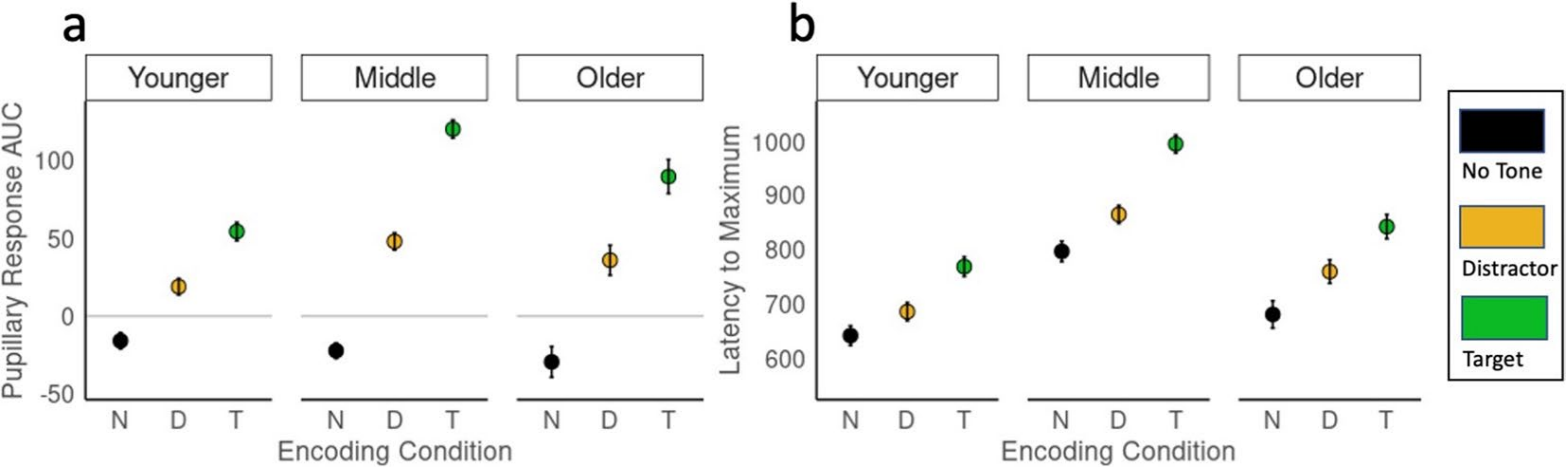
Pupillary response characteristics in response to no-tone (N, black), distractor (D, orange), and target (T, green) trials in younger, middle aged, and older adults. **a** Area under the curve (AUC), **b** maximum pupil diameter, **c** latency to maximum pupil diameter, **d** maximum positive rate of change, and **e** maximum negative rate of change. AUC data has been normalized by dynamic range; latency to maximum does not depend on normalization method. Plots show mean and standard error of the mean

Using these pupillary parameters, we constructed linear mixed effects models with age group and tone type as fixed effects, and random intercepts for image and participant. There were significant effects of tone type on both AUC and latency to maximum. ANOVAs for each model showed that all *F*(2, ~ 30,000) > 13, all *p* < 0.0001. In all age groups, pupillary responses to target tones were larger, with a longer latency to maximum, than responses to distractor tones (Fig. 3) and distractor tones were larger relative to no-tone trials. There was a main effect of age group on pupil response AUC, *F*(2,65) = 7.33, *p* = 0.001. Middle-aged adults had the largest pupillary responses relative to dynamic range, almost double that of younger adults, followed by older adults, with younger adults having the smallest proportional responses. There was also a significant interaction between age group and tone type, *F*(4,35958) = 24.46, *p* < 0.0001, with significant differences between all age groups and tone types. This interaction is captured by how the clarity of differentiation between targets and distractors decreases with age, with the weakest differentiation in the older group. To further characterize the age × condition interaction, we calculated target/distractor ratios, the ratio of average target AUC to average distractor AUC, for each individual (Fig. 2B). Linear mixed effects model on these difference scores with fixed effects of age group and random effects of participant revealed significant effects of age group on target-distractor differentiation (*F*(2,9.99) = 8724 *p* < 0.0001). There were trends toward significant effects (0.05 < *p* < 0.1) of age group on latency to maximum pupil diameter, with larger peak amplitudes associated with longer latency in the middle-aged group. Pupil response characteristics for each age group are presented in Supplementary Figs. 4, 5, 6, and 7.

#### Relationship between pupillary responses and behavioral measures

Our hypothesis was that there would be substantial independence of pupillary responses from behavioral performance. While there is evidence that pupillary responses may be associated with behavioral latency [28], our data demonstrate robust responses to distractor tones that occur without any behavioral response, consistent with pupillary responses reflecting covert central orienting in the absence of an overt manual response. Nevertheless, to investigate this, we constructed a linear mixed effects model with pupil AUC as the dependent variable and response time and age group as fixed effects and participant and image as random effects. At the level of individual trials, we found a significant relationship between target response time and pupil response AUC, *F*(1,6395) = 37.70, *p* < 0.0001, with greater pupillary response associated with longer response times and potentially cognitive effort. This relationship remained significant and in the same direction when looking only at typical response times between 200 and 600 ms but was greatly diminished, *F*(1,6536) = 6.47, *p* = 0.01. However, there was no significant effect of age group and no significant interaction between age group and response time (Supplementary Fig. 8). While slower response times are associated with larger pupillary responses, the oldest adults had both the slowest response times and smaller pupillary responses. This analysis confirms the effects of age on pupillary responses reflect changes in processing that are independent of response variables. Detailed model results can be seen in Supplementary Table 3.

In addition, we performed analyses of individual differences across the age spectrum in cognitive measures (task accuracy, average RT, MOCA, trails B) and their relation to pupillary responses. The correlation table (Table 2) reveals substantial independence of pupillary response as a covert marker of aging independent of other overt measures.

**Table 2:**
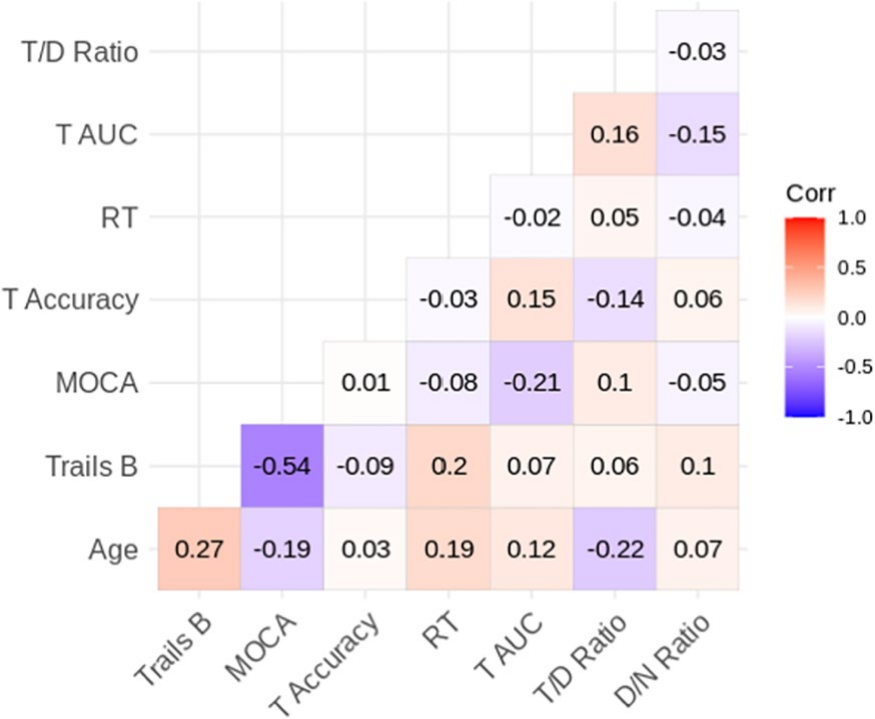
Correlation matrix showing Pearson correlation coefficient between pupil and behavioral metrics for each individual. T/D ratio is the ratio between each individual’s average target response AUC and average distractor response AUC. D/N ratio is the ratio between each individual’s average distractor response AUC and average no-tone response AUC. T AUC is average target AUC. RT is average response time to targets. T accuracy is the proportion of target trials correctly identified. MOCA is score out of 30 on the MOCA screening instrument. Trails B is the Trail Making Test Part B time in seconds. This analysis used dynamic range normalized data

**Fig. 4:**
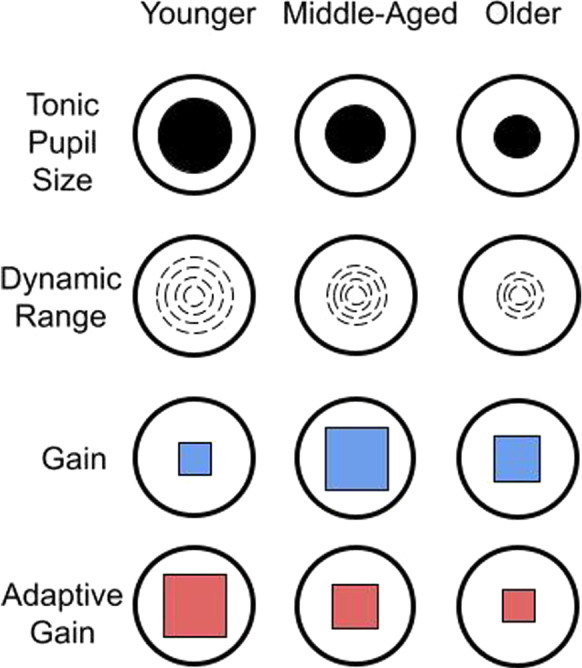
Summary figure illustrating age-related changes in pupillary orienting. Tonic (baseline) pupil size and dynamic range of pupillary orienting response both decrease across the lifespan. A nonlinear change in pupillary response gain compensates for this loss in middle aged adults, until gain is no longer sustainable in older age, resulting in diminished adaptive gain to behaviorally relevant stimulus events. Dynamic range is the 98% range of pupillary size fluctuation across the experimental session. Gain (blue squares) is the proportion of dynamic range used for target responding. Adaptive gain (red squares) is the ratio between behaviorally relevant target and behaviorally irrelevant distractor pupillary responses, which decreases in middle aged adults and decreases further in older adults

In combination, these results suggest pupillary orienting responses provide a sensitive and covert marker of age-related changes in central status relative to other traditional overt performance measures of cognitive aging. Our results demonstrate, in summary, that both baseline pupil size and pupillary dynamic range decrease with age. Gain increases in middle aged adults and decreases again in older adults. Adaptive gain, the ratio between target responses and distractor responses, is greatest in youngest adults and decreases with age, even despite increased gain in middle age (Fig. 4). We conclude from this pattern that increased gain in middle age is compensatory, supporting continued enhanced discrimination of targets from distractors, i.e., adaptive gain, but the compensatory gain response is not sustained in older adults, resulting in reduced adaptive gain and the ability to orient attention only to behaviorally relevant stimuli.

## Discussion

While behavioral task performance was largely equivalent between age groups, this study demonstrated that robust task-evoked pupillary responses (TEPRs) changed across the lifespan. All three age groups showed clear differentiation of phasic pupillary responses to behaviorally relevant target tones and behaviorally irrelevant distractor tones, consistent with action of the brainstem in the autonomic regulation of pupil size related to orienting. Even though pupil diameter increased for both distractor and target tones, it was greatest for target tones. As expected, the age groups differed in their baseline pupil diameter, and both pupil diameter and pupillary dynamic range decreased with age. These age differences in pupillary responses reflected a marked nonlinear progression across the lifespan; middle aged adults demonstrated increased amplitude of phasic responding compared to young adults, which then diminished in older adults. This nonlinear pattern, specifically for pupillary orienting, suggests that compensatory activity in the LC present during middle age may be unsustainable in old age prior to changes in neuropsychological status.

We sought to define putative transitions in brainstem function across the lifespan through pupillary dynamics. Age group definitions were based on a grid search using a summary score resulting from principal component analysis of 6 metrics of a random 10% of target trials. We then further characterized pupillary dynamics in the remaining 90% of trials. This approach allowed a bottom-up data-driven specification of the boundaries of age bands, resulting in the best fit over 3 broad age groups. Middle age was defined using this method as being 42–68 years of age. While this age grouping is nontraditional, our method may allow us to more accurately depict how pupillary responses, and their putative brainstem origins, change with age. This may contribute to the observed heterogeneity in characterization of pupillary responses across lifespan in published literature [17, 28, 36, 55, 76], where studies tend to compare younger and older groups.

Our deeper characterization of pupillary dynamic features demonstrated that baseline pupil diameter and orienting response size (AUC) follow different trajectories across the lifespan. Baseline pupil diameter decreases by middle age, while AUC increases temporarily in middle age. Middle aged adults use on average almost twice as much of their dynamic range of pupillary orienting to targets as younger adults. This suggests that even after pupil diameter is limited by lifespan developmental changes, whether they be peripheral or central, middle aged adults compensate, likely with increased engagement of the LC and related systems, to adaptively increase gain [2] as a source of cognitive reserve [14, 52, 57] to help maintain behavioral performance. This is not the case with older adults, who, while continuing to use more of their dynamic range than younger adults, no longer showed the additional gain supporting increased amplitude of pupillary responses that was found in middle age.

Looking at pupillary response profiles, distractor responses were virtually identical between middle-aged and older adults, but older adults had smaller target responses. Thus while overall pupil responses of older adults reversed in magnitude after middle age, appearing more like younger individuals, their orienting profile was distinct, with significantly reduced adaptive gain. Decreased differentiation of target from distractor responses therefore characterizes older adults and differentiates them from the young adults. In combination, these data support both the LC cognitive reserve theory [52] and adaptive gain [1]. Consistent with the cognitive reserve theory, the data revealed compensatory increases in pupillary responsiveness in middle-aged adults that allowed them to produce responses similar to those of young adults, while older adults may no longer be able to fully compensate. Consistent with LC adaptive gain theory, older adults’ reversal in the magnitude of pupillary orienting to targets suggests an impaired underlying capacity for the LC to provide adaptive gain for the most behaviorally relevant events [2]. 

The cause of decreased dynamic range in middle-aged and older adults is unclear. We count at least 3 possible origins of the age-related miosis, a phenomenon which itself has been known for decades [45]: (1) It could result from a decrease in the contractility of the dilator pupillae; (2) from reduced sensitivity to the neurochemical signals from central processes that regulate pupil diameter; or (3) from changes in the central processes themselves, whether that be reduced activity in brainstem nuclei or altered distribution of the resulting neurotransmitters. Decreased dynamic range in midlife did not diminish much further in older age. This suggests the origins of small baseline pupils and restricted dynamic maybe be compensatory, e.g., decreased age-related optic aberrations, but were independent from the underlying neurocognitive changes between middle and older ages that were reflected in marked differences in pupil dynamics.

Our results themselves suggest that the lifespan differences we have observed are not the result of peripheral muscular weakness. Our middle-aged adults have an increased rate of change and an increased absolute response size despite smaller dynamic range and smaller baseline pupil diameter. If peripheral muscular weakness were the cause of our observed age differences, then we would expect rate of change to decline with baseline pupil size. However, our supplementary data demonstrates that middle-aged adults have increased absolute and relative rate of change, despite significantly smaller tonic pupil size and dynamic range. Even though reduced muscular contractility has been considered a possible explanation for senile miosis for many years [45], there is evidence to the contrary showing that application of sympathomimetic drugs directly to the eye results in equivalent or enhanced pupillary responses in older adults, rather than diminished ones [37], in line with our results. Additionally, following trial onset (of each image), pupil diameter begins to increase at the same time or even earlier for older adults than for younger adults, close to 400 ms in all three groups. Since the earliest pupillary response to light is approximately 200 ms, this leaves 200 ms for auditory cortical processing and engaging the pupillary response, consistent with an early N100 auditory modulation [19]. Overall, our results suggest that the observed age differences in pupillary dynamics during orienting to behaviorally relevant events are very likely to arise from changes in central control, making them more useful as a potential peripheral biomarker of central aging. A major future challenge remains in identifying which aspects of age differences in pupillary orienting responses are associated with distinct central origins.

Age-related miosis might reflect a paradoxical downstream consequence of increased compensatory tonic noradrenergic signaling in the brainstem [38]. For example, compensatory upregulation in noradrenergic signaling has been observed in surviving LC neurons following neurodegenerative damage to the LC [34, 47, 69]. According to the adaptive gain theory of LC function [3] and published experiments [32], increased tonic norepinephrine (NE) release may reduce the ability of the system to respond in a phasic manner. Therefore, it may be important to know what proportion of available range is being used in a pupillary response, rather than simply the size of the response, since the available range may be limited by the tonic firing rate. Continuing to assess dynamic range may allow future studies to interpret the meaning of each individual’s pupil response more precisely, especially as the causes of age-related miosis are further investigated. For example, we hypothesize based on our results that individuals with reduced baseline pupil size but increased phasic responding are in the “successful compensation” phase of development, while those who have reduced baseline pupil size and decreased phasic responding are in the “unsuccessful compensation” phase. Our results thus provide support for the theory that the function of the noradrenergic system is critical for maintaining cognitive functions in older adults.

Across the lifespan, the pupil story is complex. Middle-aged adults have reduced pretrial baseline diameter compared with younger adults, which could indicate decreased tonic NE release, and also correspondingly larger pupillary orienting. However, older adults have the smallest pretrial baseline diameter but also reduced pupillary orienting to targets, compared with middle-aged adults and thus is very unlikely to be caused simply by a change in tonic NE release. There is something else at play. Since identification of salient information is understood to be a major function of the noradrenergic system [77, 78], the changes we observed in older adults could be important for understanding the effects of aging on cognition. We know that older adults are utilizing more of their available dynamic range, but with the least amount of differentiation between target and distractor types. What is responsible for this?

A 2016 meta-analysis of behavioral orienting responses in older adults paints a complicated portrait of orienting in the aging brain, with many faculties fully intact and others diminished, and argues that inconsistencies in the literature on orienting responses could be partly due to a failure to account for age-related changes in noradrenergic signaling [21]. A recent study showed that while older adults had an enhanced alerting response compared to younger adults under some conditions, this effect was absent in older adults with mild cognitive impairment [35]. Overall, the literature mirrors our findings, in that there are clear age differences in attending and orienting, but these responses are not simply diminished in older adults. Compensatory activity in stimulus processing to account for age-related deficits may be a common feature of the aging process [21]. More work is needed to fully understand the nature of orienting changes in older adults, and whether they stem from changes in the noradrenergic system. This is crucial to an understanding of the LC to healthy and pathological aging because the LC is one of the first pathological targets of Alzheimer’s disease. The evidence described in this paper, points to the presence of compensatory upregulation of attention and orienting functions, especially in midlife, underscores its importance in untangling the earliest precursors of disease.

In terms of limitations, while researchers use pupillary responses as an assessment of LC function [20, 24, 54] it should be noted that they are correlated with activity in more than one brain region [16, 44, 53], and as such this study is unable to directly link the observed changes to the LC recruitment or noradrenergic signaling. However, the results certainly suggest greater engagement, on some level, of bottom-up orienting pathways in middle-aged and older adults, reflected in peripheral autonomic control of the pupil. A further limitation of this study was the fact that our pupillometric measurements took place while participants were lying down during MRI acquisition, which enhances parasympathetic tone. The effects of this body position on autonomic responses, which may including pupillary dynamics, may also have differential effects on younger and older adults [15]. Also, to ensure that participants could distinguish between target and distractor tones during our task, it was necessary to increase the sound level of the tones to make them detectable for some participants in the older group, especially those who were hearing aid users. Some previous work has shown that pupillary responses are strongly influenced by perceived loudness [48]. Critically, the target and distractor tones in our task were equivalent in sound pressure level yet resulted in very different pupillary responses. Finally, while the target tones required a motor response in this experiment, our previous work has demonstrated that differences in pupillary responses are present even without a motor response [66, 67]. The oldest adults also had the longest behavioral response to targets, but the smallest pupillary response, inconsistent with a large role for motor planning or execution. Given its small size and deep brainstem location it is challenging, but possible, to measure the LC directly with advanced neuroimaging techniques [73]. Future studies will examine the relationship between these age-related changes to pupillary dynamics and LC structure and function across the lifespan.

## Conclusions

We showed that in contrast to monotonic decreases in tonic pupillary diameter, phasic pupillary responses to behaviorally relevant events varied nonlinearly across the lifespan, with a peak in middle age, consistent with age-related changes in brainstem regions related to orienting. In light of the relationship between orienting and the LC and noradrenergic system, and the LC’s importance early in the process of neurodegeneration, cognitive regulation of the pupil is a promising window into age-related changes in brainstem function before any behavioral signs emerge.

### Supplementary Information

Below is the link to the electronic supplementary material.

Supplementary file1 (PDF 0.98 MB)

## Data Availability

Data (R dataframe of extracted pupil metrics and raw pupil data) available from Open Science Framework https://osf.io/p6ux5/.
