# Supplementary material for: Nonlinear changes in pupillary attentional orienting responses across the lifespan
Source: GeroScience. 2023 Jun 15;46(1):1017–33. doi: 10.1007/s11357-023-00834-1 (PMC10828243; doi:10.1007/s11357-023-00834-1)
Supplement: Supplementary file 1 — Supplementary file1 (PDF 0.98 MB) [file 11357_2023_834_MOESM1_ESM.pdf]

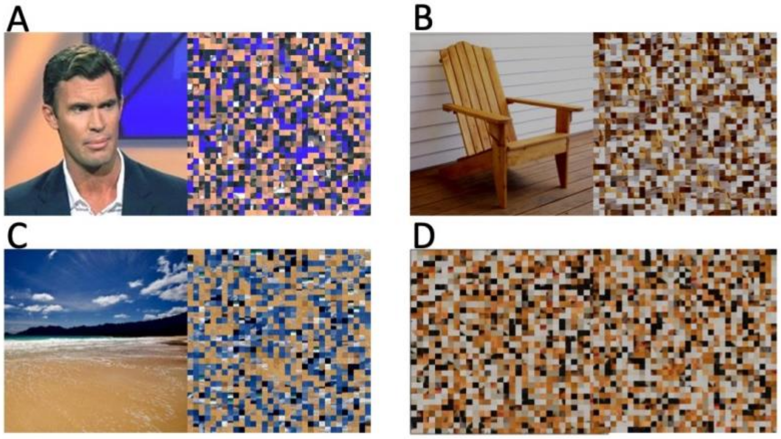

Supplementary Fig. 1. Examples of task stimuli. (A) Male face image and its scrambled mask. (B) Chair image and its scrambled mask. (C) Beach image and its scrambled mask. (D) Scrambled image for non-task trials (drawn from pool of male faces) and a re-scrambled mask. Female faces, cars and forest images not shown. For task trials, a non-scrambled image was shown for 650 ms followed by a scrambled version of the same image for 650 ms. For non-task trials, a scrambled image (drawn from pool of male faces) was shown for 650 ms followed by a re-scrambled version of the same image for 650 ms.

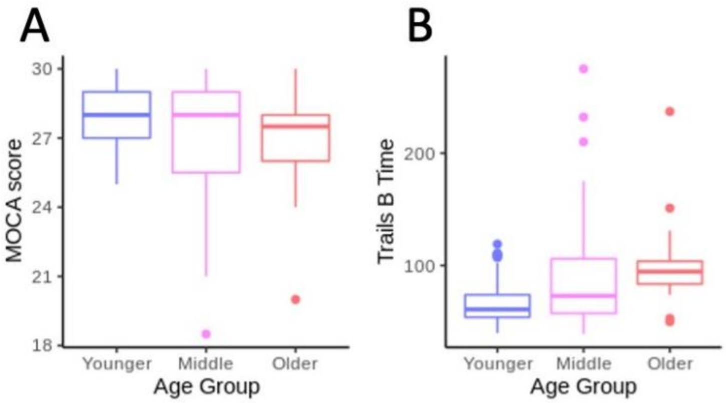

Supplementary Fig. 2. Neurocognitive screening results. (A) Average Montreal Cognitive Assessment (MOCA) scores in each age group. (B) Average time to complete Trail Making Test Form B (Trails B). The top and bottom of the box show 25th and 75th percentile with the mean indicated by the middle line. Whiskers show smallest and largest non-outlier values, individual points indicate outliers.

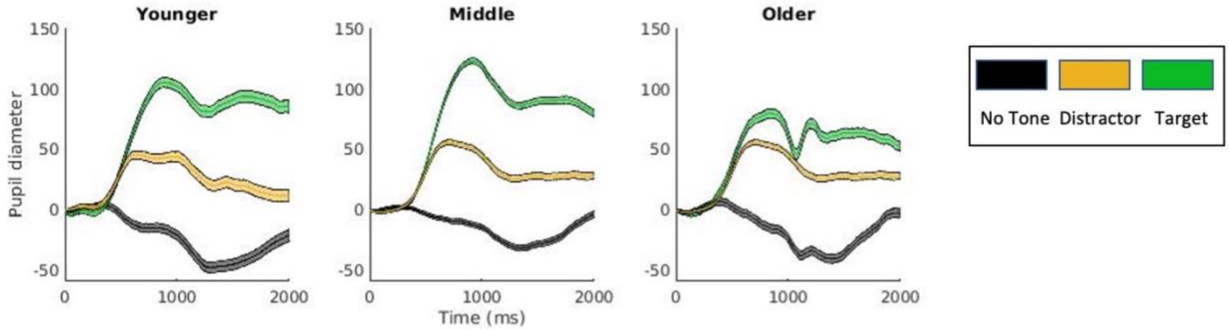

Supplementary Fig. 3. Pupillary response curves in response to no-tone (N, black), distractor (D, orange) and target (T, green) trials in younger, middle aged and older adults after pretrial baseline normalization only. In all plots, the colored ribbon shows standard error of the mean.

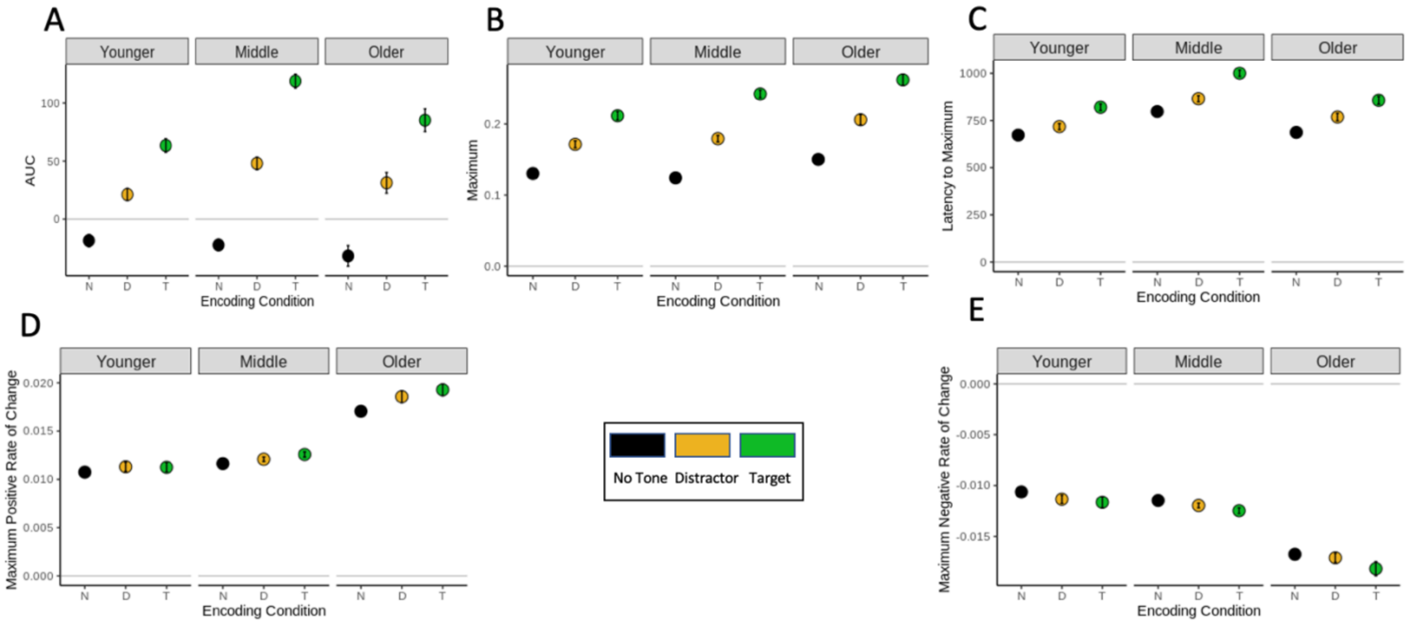

Supplementary Fig. 4. Pupillary response characteristics in response to no-tone (N, black), distractor (D, orange) and target (T, green) trials in younger, middle aged and older adults. (A) Area under the curve (AUC), (B) Maximum pupil diameter, (C) Latency to maximum pupil diameter, (D) maximum positive rate of change and (E), maximum negative rate of change. All data has been normalized by dynamic range except for latency to maximum which does not depend on normalization method. All plots show mean and standard error of the mean.

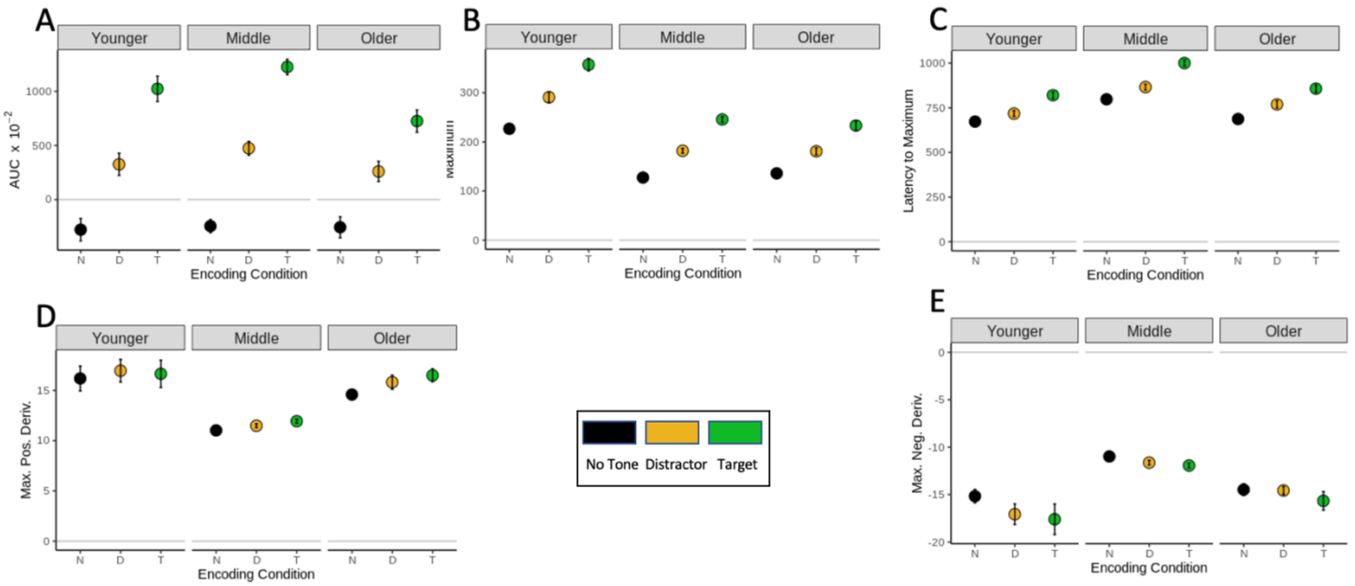

Supplementary Fig. 5. Pupillary response characteristics in response to no-tone (N, black), distractor (D, orange) and target (T, green) trials in younger, middle aged and older adults. (A) Area under the curve (AUC), (B) Maximum pupil diameter, (C) Latency to maximum pupil diameter, (D) maximum positive rate of change and (E), maximum negative rate of change. All data has been normalized by pretrial baseline only, except latency to maximum which does not depend on normalization. All plots show mean and standard error of the mean.

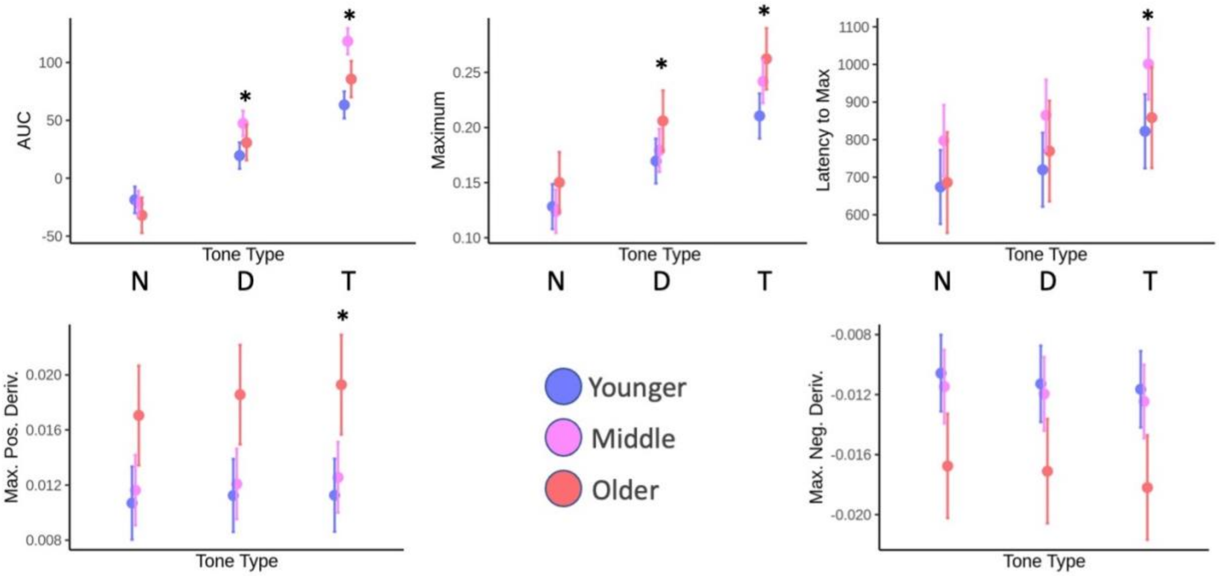

Supplementary Fig. 6. Modeled estimates for five pupil characteristics (AUC, Maximum, Latency to Maximum, Maximum Positive Rate of Change, Maximum Negative Rate of Change) by tone type (N = No Tone, D = Distractor, T = Target) and age group (blue = younger, red = older) after fitting with the models described in Table 1. All data is normalized by dynamic range. Asterisks indicate significant age*tone type interactions. Error bars show 95% confidence interval.

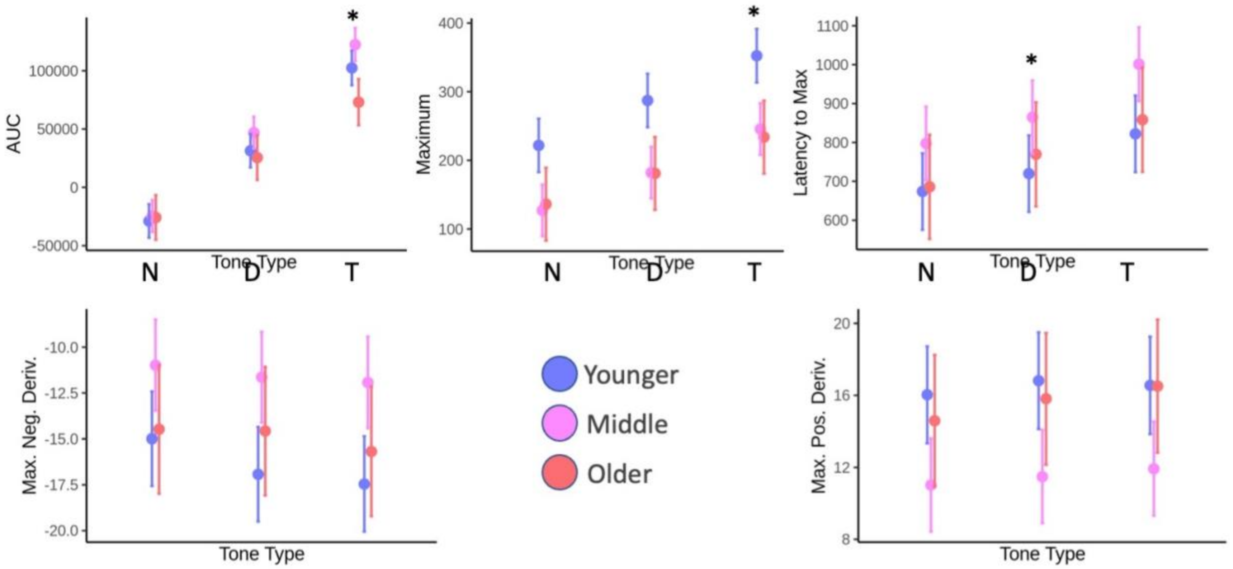

Supplementary Fig. 7. Modeled estimates for five pupil characteristics (AUC, Maximum, Latency to Maximum, Maximum Positive Rate of Change, Maximum Negative Rate of Change) by tone type (N = No Tone, D = Distractor, T = Target) and age group (blue = younger, red = older) after fitting with the models described in Table 1. All data is normalized by pretrial baseline only. Asterisks indicate significant age*tone type interactions. Error bars show 95% confidence interval.

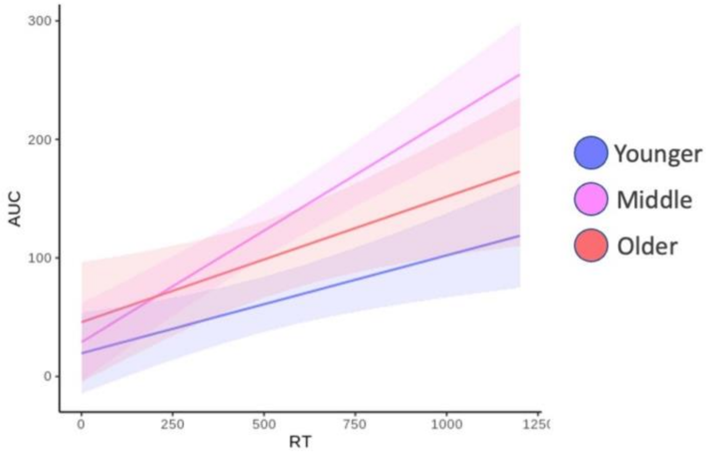

Supplementary Fig. 8. Plot shows estimated effects of reaction time (RT) on pupil AUC, from model described in Supplementary Table 3.

Supplementary Table 1. Result of linear mixed effects model on five pupil characteristics (AUC, Maximum, Latency to Maximum, Maximum Positive Derivative, Maximum Negative Derivative) with fixed effects of tone type and age group, and random effects of image and participant. These models use dynamic range normalized data.

Effect of Age Group and Tone on Pupil Variables

	AUC				Maximum				Latency to Max				Max. Pos. Deriv.				Max. Neg. Deriv.			
Predictors	Estimates	CI	Statistic	p	Estimates	CI	Statistic	p	Estimates	CI	Statistic	p	Estimates	CI	Statistic	p	Estimates	CI	Statistic	p
Intercept	-18.67	-30.07 – -7.27	-3.21	**0.004**	0.13	0.11 – 0.15	12.38	**<0.001**	673.88	575.52 – 772.24	13.43	**<0.001**	0.01	0.01 – 0.01	7.90	**<0.001**	-0.01	-0.01 – -0.01	-8.12	**<0.001**
Dist. Tone	38.22	29.99 – 46.44	9.11	**<0.001**	0.04	0.03 – 0.05	11.89	**<0.001**	45.87	23.50 – 68.25	4.02	**<0.001**	0.00	0.00 – 0.00	2.39	0.084	-0.00	-0.00 – -0.00	-3.27	**0.008**
Targ. Tone	82.09	73.48 – 90.69	18.70	**<0.001**	0.08	0.08 – 0.09	22.59	**<0.001**	148.24	124.80 – 171.68	12.39	**<0.001**	0.00	0.00 – 0.00	2.35	0.084	-0.00	-0.00 – -0.00	-4.70	**<0.001**
Age Middle	-3.17	-18.52 – 12.18	-0.40	0.686	-0.00	-0.03 – 0.02	-0.30	0.766	123.43	-13.00 – 259.86	1.77	0.305	0.00	-0.00 – 0.00	0.50	1.000	-0.00	-0.00 – 0.00	-0.50	1.000
Age Older	-13.34	-32.00 – 5.33	-1.40	0.323	0.02	-0.01 – 0.06	1.26	0.418	11.93	-154.11 – 177.97	0.14	0.888	0.01	0.00 – 0.01	2.78	**0.038**	-0.01	-0.01 – -0.00	-2.81	**0.030**
Dist. Tone*Age Middle	31.00	19.88 – 42.13	5.46	**<0.001**	0.01	0.00 – 0.02	2.96	**0.012**	21.62	-8.91 – 52.14	1.39	0.495	-0.00	-0.00 – 0.00	-0.31	1.000	0.00	-0.00 – 0.00	0.74	1.000
Targ. Tone*Age Middle	58.19	46.51 – 69.88	9.76	**<0.001**	0.04	0.03 – 0.05	7.18	**<0.001**	55.72	23.64 – 87.80	3.40	**0.004**	0.00	-0.00 – 0.00	1.09	0.832	0.00	-0.00 – 0.00	0.26	1.000
Dist. Tone*Age Older	24.57	11.10 – 38.04	3.57	**0.001**	0.01	0.00 – 0.03	2.53	**0.034**	37.73	0.73 – 74.73	2.00	0.228	0.00	0.00 – 0.00	2.47	0.082	0.00	-0.00 – 0.00	0.99	1.000
Targ. Tone*Age Older	35.61	21.45 – 49.77	4.93	**<0.001**	0.03	0.02 – 0.04	4.98	**<0.001**	24.29	-14.60 – 63.19	1.22	0.495	0.00	0.00 – 0.00	4.07	**<0.001**	-0.00	-0.00 – 0.00	-0.96	1.000
ICC	0.03				0.09				0.18								0.28			
Observations	36425				36425				36425				36425				36425			
Marginal R^2 / Conditional R^2	0.053 / 0.078				0.059 / 0.146				0.026 / 0.197				0.056 / NA				0.035 / 0.301			

Supplementary Table 2. Result of linear mixed effects model on five pupil characteristics (AUC, Maximum, Latency to Maximum, Maximum Positive Derivative, Maximum Negative Derivative) with fixed effects of tone type and age group, and random effects of image and participant. These models use pretrial baseline normalization only.

Effect of Age Group and Tone on Pupil Variables

	AUC				Maximum				Latency to Max				Max. Pos. Deriv.				Max. Neg. Deriv.			
Predictors	Estimates	CI	Statistic	p	Estimates	CI	Statistic	p	Estimates	CI	Statistic	p	Estimates	CI	Statistic	p	Estimates	CI	Statistic	p
Intercept	-28841.78	-43181.76 – -14501.79	-3.94	**0.001**	221.72	182.74 – 260.71	11.15	**<0.001**	673.88	575.52 – 772.24	13.43	**<0.001**	16.03	13.34 – 18.72	11.68	**<0.001**	-14.99	-17.57 – -12.42	-11.41	**<0.001**
Dist. Tone	60196.62	48156.41 – 72236.84	9.80	**<0.001**	65.48	55.05 – 75.90	12.31	**<0.001**	45.87	23.50 – 68.25	4.02	**<0.001**	0.78	-0.28 – 1.85	1.44	0.989	-1.93	-2.94 – -0.93	-3.77	**0.001**
Targ. Tone	131233.67	118632.43 – 143834.90	20.41	**<0.001**	130.65	119.74 – 141.57	23.46	**<0.001**	148.24	124.80 – 171.68	12.39	**<0.001**	0.52	-0.60 – 1.64	0.91	1.000	-2.47	-3.52 – -1.41	-4.58	**<0.001**
Age Middle	4277.16	-15072.84 – 23627.16	0.43	1.000	-94.70	-148.73 – -40.68	-3.44	**0.003**	123.43	-13.00 – 259.86	1.77	0.305	-5.02	-8.75 – -1.28	-2.63	0.068	4.01	0.43 – 7.58	2.20	0.167
Age Older	3037.30	-20480.87 – 26555.47	0.25	1.000	-85.58	-151.33 – -19.84	-2.55	**0.043**	11.93	-154.11 – 177.97	0.14	0.888	-1.45	-5.99 – 3.09	-0.62	1.000	0.51	-3.83 – 4.86	0.23	0.818
Dist. Tone*Age Middle	11280.28	-5057.56 – 27618.12	1.35	0.704	-10.40	-24.58 – 3.79	-1.44	0.222	21.62	-8.91 – 52.14	1.39	0.495	-0.32	-1.78 – 1.15	-0.42	1.000	1.28	-0.10 – 2.66	1.81	0.210
Targ. Tone*Age Middle	15747.72	-1416.84 – 32912.27	1.80	0.361	-12.12	-27.02 – 2.79	-1.59	0.222	55.72	23.64 – 87.80	3.40	**0.004**	0.39	-1.15 – 1.93	0.49	1.000	1.53	0.07 – 2.98	2.05	0.167
Dist. Tone*Age Older	-8910.83	-28706.01 – 10884.35	-0.88	1.000	-20.67	-37.87 – -3.48	-2.36	0.055	37.73	0.73 – 74.73	2.00	0.228	0.44	-1.34 – 2.22	0.49	1.000	1.84	0.16 – 3.52	2.15	0.167
Targ. Tone*Age Older	-32375.76	-53180.39 – -11571.13	-3.05	**0.014**	-33.02	-51.09 – -14.95	-3.58	**0.002**	24.29	-14.60 – 63.19	1.22	0.495	1.41	-0.47 – 3.28	1.47	0.989	1.26	-0.50 – 3.03	1.40	0.323
ICC	0.02				0.14				0.18								0.06			
Observations	36425				36425				36425				36425				36425			
Marginal R^2 / Conditional R^2	0.032 / 0.048				0.059 / 0.186				0.026 / 0.197				0.008 / NA				0.008 / 0.069			

Supplementary Table 3. Result of linear mixed effects model on phasic responding (AUC) with fixed effects of reaction time and age group, and random effects of image and participant.

Effect of Age Group and RT on AUC

	AUC			
Predictors	*Estimates*	*CI*	*Statistic*	*p*
Intercept	38712.26	-9181.96 – 86606.48	1.58	0.453
RT	126.86	47.77 – 205.95	3.14	**0.010**
Age Middle	-34002.74	-100930.20 – 32924.71	-1.00	0.958
Age Older	-16083.07	-100925.62 – 68759.47	-0.37	1.000
RT * Age Middle	120.18	7.57 – 232.80	2.09	0.182
RT * Age Older	-1.78	-139.35 – 135.78	-0.03	1.000
ICC	0.07			
Observations	8712			
Marginal R^2 / Conditional R^2	0.009 / 0.075			
